# Visual snow syndrome and the emperor’s new clothes

**DOI:** 10.1093/braincomms/fcac178

**Published:** 2022-07-06

**Authors:** Daniel Kondziella

**Affiliations:** Department of Neurology, Rigshospitalet, Copenhagen University Hospital, Copenhagen, Denmark; Department of Clinical Medicine, University of Copenhagen, Copenhagen, Denmark

## Abstract

This scientific commentary refers to ‘Microstructure in patients with visual snow syndrome: an ultra-high field morphological and quantitative MRI study’, by Strik *et al*. (https://doi.org/10.1093/braincomms/fcac164)

**This scientific commentary refers to ‘Microstructure in patients with visual snow syndrome: an ultra-high field morphological and quantitative MRI study’, by Strik *et al*. (** https://doi.org/10.1093/braincomms/fcac164 **)**

The term ‘visual snow syndrome’ was coined in 2013^1^ and denotes a chronic condition associated with the continuous perception of tiny flickering spots throughout the visual field, resembling the static noise of an untuned television or snow in the air (Fig. 1). In addition, photophobia, palinopsia, and blue field entoptic phenomena can occur, and competing neurological and ophthalmological disorders must be absent.^2,3^ Operational criteria were introduced in 2017 and 2018.^3,4^ Attempts of medical treatment have failed.^5^

**Figure 1:**
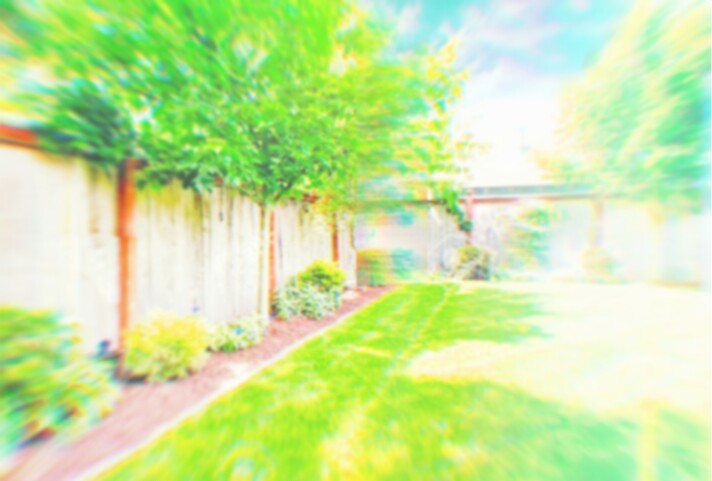
**Illustrative image of visual snow syndrome.** Illustrative image provided by a person with visual snow syndrome and visual migraine aura, reproduced with permission.

The biological mechanisms of visual snow syndrome are poorly understood. Most data come from isolated case reports, smaller case series^2,3^ or surveys based on self-help groups.^6^ However, increasing scientific publications, wide media coverage, and extensive activity on social platforms are evidence of growing awareness of visual snow syndrome (Fig. 2). Every now and then, I see a person with visual snow syndrome in my outpatient neurology service who has been referred for concerns after researching dedicated internet websites.^7,8^ Almost invariably, these people leave the consultation relieved after being reassured that visual snow syndrome is a harmless condition.

**Figure 2:**
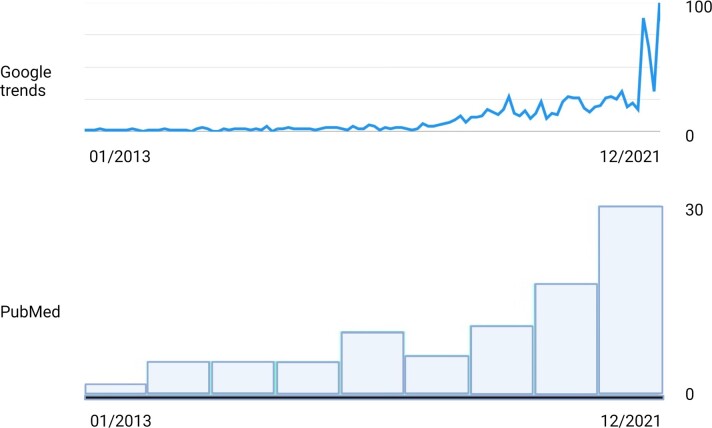
**Visual snow syndrome searches and citations.** Visual snow syndrome, the term of which was introduced in 2013, is an increasingly popular entity with both lay people and academics, as illustrated by Google searches (above; Google Relative Search Volumes, ranging from 0 to 100) and PubMed citations (below, ranging from one article in 2013 to 31 articles in 2021).

In this issue of *Brain Communications*, Strik *et al*.^9^ provide data from a 7 Tesla MRI structural neuroimaging study on 40 people with visual snow syndrome and 43 controls, roughly matched for age and sex. Their key message is that while morphometry was unchanged, they observed ‘widespread changes in grey matter microstructure, which followed a caudal–rostral pattern and affected the occipital cortices most profoundly.’ The technical aspects of the MRI study and the statistics are sound, and the results, although not unexpected, are interesting. However, there are concerns related to the recruitment of study subjects, the interpretation of the data, and the overarching conclusions.

As expected from a condition without a clinically identifiable structural cause, most findings were correlations and trends that did not survive correction for multiple comparisons. Unfortunately, the interpretation of the few positive findings appears much overstretched. In my opinion, the conclusion that these data ‘(contribute) significantly to our understanding of visual snow syndrome’ is not warranted.

Although visual snow syndrome was unheard of until very recently, its prevalence in the UK general population has been estimated to be 2%, with another 2% having visual snow without meeting the criteria for visual snow syndrome.^10^ This suggests that worldwide there are probably tens or hundreds of millions of people who are unaware that they have this condition and who are not complaining either. However, Stik *et al*. recruited self-elected people via online, print and media advertisements, which results in a fundamental bias, because these people come from a tiny fraction of the population with visual snow who is not only aware of the condition but also sufficiently concerned or interested to participate in the study. This tiny fraction is simply not representative for the vast majority with visual snow.

For the reasons outlined, I strongly oppose the authors’ notions that ‘it is clear that visual snow syndrome is a disorder of the central nervous system’, that there is ‘pathology’ associated with it, that people with visual snow syndrome are ‘patients’, and that there is a need for ‘new treatment strategies’.^9^

In fact, visual snow syndrome is a harmless physiological phenomenon, whose existence has gone undetected until a few years ago.^1^ This, and the relatively high prevalence of visual snow syndrome in the background population,^10^ render visual snow syndrome incompatible with a progressive brain disorder. Consistent with this is the observation that the mean age of those with visual snow syndrome is much higher in unprimed laypersons^10^ than reported in previous case series,^2,3^ suggesting that younger people are more likely to seek medical advice or register in internet-based support groups, although they are less commonly affected.

There is therefore absolutely no need to ascribe visual snow syndrome any pathological value. To the contrary, statements like the ones cited here are doing a disservice to the very few people who are concerned that they could have a neurological disorder of unknown aetiology and without treatment options. What these people really need is reassurance that visual snow syndrome is entirely benign and nothing to worry about. This is particularly important given the association of visual snow syndrome with depression and anxiety.^2,3,5,10^

In summary, the technical aspects of the article are well done, but the data are flawed owing to recruitment bias and the conclusions drawn are not warranted. The problem is that even though a few microstructural changes could be demonstrated; this is not proof that visual snow syndrome is a ‘disease’. Rather, it is proof that 7 Tesla MRI is an enormously sophisticated technology that likely will identify microstructural changes in a variety of physiological conditions in the future. A condition that is present in 2–4% of the general population and that has completely gone under the radar until a few years ago, is a harmless physiological phenomenon. From a clinical neurologist’s perspective, the intention to brand it as a ‘disease’ is unfortunate. When it comes to the recent hype around visual snow syndrome, I am reminded of the famous fairytale by H.C. Andersen: The emperor is naked.

## Data Availability

Data sharing is not applicable to this article as no new data were created or analysed.
